# Inflammation and neuronal death in the motor cortex of the wobbler mouse, an ALS animal model

**DOI:** 10.1186/s12974-015-0435-0

**Published:** 2015-11-24

**Authors:** Carolin Dahlke, Darius Saberi, Bastian Ott, Beate Brand-Saberi, Thomas Schmitt-John, Carsten Theiss

**Affiliations:** Department of Cytology, Institute of Anatomy, Ruhr University Bochum, Universitätsstraße 150, 44780 Bochum, Germany; Department of Molecular Biology and Genetics, Molecular Cell and Developmental Biology, University of Aarhus, C.F. Møllers Allé 3, 8000 Aarhus, Denmark; Department of Anatomy and Molecular Embryology, Institute of Anatomy, Ruhr University Bochum, Universitätsstraße 150, 44780 Bochum, Germany

**Keywords:** Amyotrophic lateral sclerosis, Wobbler mouse, Inflammation, Neurodegeneration, Microglial cells, Astrocytes, Caspase 3, Tumor necrosis factor α

## Abstract

**Background:**

Amyotrophic lateral sclerosis (ALS) is a fatal neurodegenerative disorder of the upper and lower motor neurons, characterized by rapid progressive weakness, muscle atrophy, dysarthria, dysphagia, and dyspnea. Whereas the exact cause of ALS remains uncertain, the wobbler mouse (phenotype WR; genotype *wr/wr*) equally develops a progressive degeneration of motor neurons in the spinal cord and motor cortex with striking similarities to sporadic human ALS, suggesting the possibility of a common pathway to cell death.

**Methods:**

With the aid of immunohistochemistry, confocal laser scanning microscopy, and transmission electron microscopy techniques, we analyze the proliferation behavior of microglial cells and astrocytes. We also investigate possible motor neuron death in the mouse motor cortex at different stages of the wobbler disease, which so far has not received much attention.

**Results:**

An abnormal density of Iba-1-positive microglial cells expressing pro-inflammatory tumor necrosis factor (TNF) alpha- and glial fibrillary acidic protein (GFAP)-positive activated astroglial cells was detected in the motor cortex region of the WR mouse 40 days postnatal (d.p.n.). Motor neurons in the same area show caspase 3 activation indicating neurodegenerative processes, which may cause progressive paralysis of the WR mice. It could also cause cell degeneration, such as vacuolization, dilation of the ER, and swollen mitochondria at the same time, and support the assumption that inflammation might be an important contributing factor of motor neuron degeneration. This would appear to be confirmed by the fact that there was no conspicuous increase of microglial cells and astrocytes in the motor cortex of control mice at any time.

**Conclusions:**

Activated microglial cells secrete a variety of pro-inflammatory and neurotoxic factors, such as TNF alpha, which could initiate apoptotic processes in the affected wobbler motor neurons, as reflected by caspase 3 activation, and thus, the neuroinflammatory processes might influence or exacerbate the neurodegeneration. Although it remains to be clarified whether the immune response is primary or secondary and how harmful or beneficial it is in the WR motor neuron disease, anti-inflammatory treatment might be considered.

**Electronic supplementary material:**

The online version of this article (doi:10.1186/s12974-015-0435-0) contains supplementary material, which is available to authorized users.

## Background

Neurodegenerative motor neuron disorders like amyotrophic lateral sclerosis (ALS) induce severe phenotypes by denervation of the skeletal muscles and lead to a rapid progressive weakness, muscle atrophy, dysarthria, dysphagia, dyspnoea and, consequentially, death. Even though there is no effective therapeutic treatment, apart from life-prolonging measures like the usage of riluzole, a significant advance has been made in the understanding of possible underlying molecular mechanisms such as oxidative stress due to mitochondrial dysfunction, protein aggregation, impaired axonal transport, the complex interplay of the several cellular effects, and neuroinflammation in ALS [1–6].

**Fig. 1:**
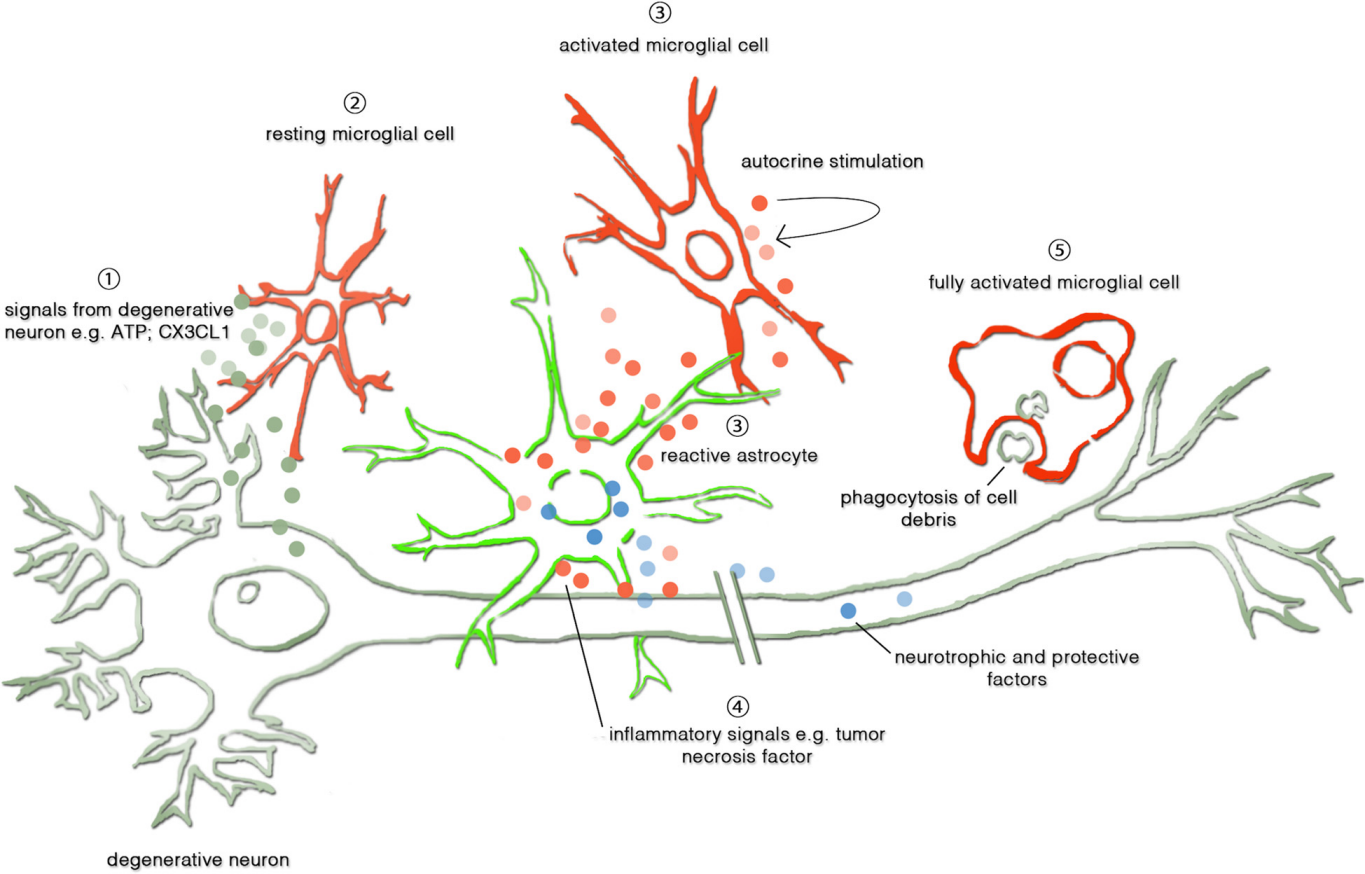
Neuroinflammatory process aging or various genetic or environmental defects may induce early damage of motor neurons. ② Resting microglial cells and astrocytes pass a gradual activation process after sensitization by signals of the affected motor neuron. ① ATP or CX3CL1 for example leads to ③ morphological changes and ④ secretion of pro-inflammatory factors like tumor necrosis factor, which is able to induce systemic inflammation, apoptotic cell death, and in the end, ⑤ phagocytosis of neuronal cell debris. In neurodegenerative diseases, activated microglial cells and reactive astrocytes engage in cytokine communication as well, while also maintaining a synergistic balance of neuroprotection and cytotoxity for as long as possible

Chronic neuroinflammation associated with neurotoxicity has been well established as one of the primary pathomechanisms in numerous neurological disorders and diseases such as Alzheimer disease (AD), Huntington disease, Parkinson disease (PD), as well as ALS. Inflammation describes the host of cellular and molecular changes, including the induction of intracellular pathways, the release of inflammatory mediators, and the continuous activation and inclusion of glial and immune cells [7, 8]. Microglial cells are similar to peripheral macrophages and constitute the main resident immune cells of the central nervous system (CNS). Activated by cell necrosis factor, microglial cells are able to phagocytose apoptotic cells, DNA fragments, cellular debris, and plaques, after they have passed through a dramatic morphological change from anti-inflammatory and neuroprotective cells to pro-inflammatory and neurotoxic cells [9] (Fig. 1). They are known as important components in neurodegenerative dysfunctions, specifically distinguished by increasing cell loss. Beside this, activated microglial cells secrete pro-inflammatory and neurotoxic factors like tumor necrosis factor alpha (TNF-α), interleukin-1 beta (IL-1β), and free radicals [8], which are reported to be deleterious to neurons and may cause their cell death [10]. In response to signals of damaged or degenerative neurons and activated microglial cells, astrocytes also become activated and are able to hinder neuron regeneration as well. Neuroinflammation, as assumed in the wobbler mutation with regard to motor neurons, may contribute to the occurrence of neuronal dysfunction and thus leads to a number of locomotor deficits like decreased use and strength of hind- and forelimbs.

The autosomal recessive *wr/wr* mutation spontaneously occurred in a C57BL/Fa stock of mice at the Institute of Animal Genetics in Edinburgh and was first described by Falconer in 1956 [11]. The wobbler mutation was linked to motor neuron degeneration [12] and constitutes an animal model for sporadic ALS. It was mapped to the proximal end of mouse chromosome 11 [13], leading to the identification of the affected gene: the vacuolar-vesicular protein sorting factor 54 (Vps54) [14]. Vps54 is a component of the multi-subunit Golgi-associated retrograde protein (GARP) complex, a tethering complex that attaches endosome-derived transport vesicles to the trans-Golgi network [15]. The wobbler point mutation of leucine-967 to glutamine causes a destabilization of Vps54 protein and thereby the GARP complex [15]. The decreased GARP stability leads to impaired retrograde vesicle traffic and enlarged endosomal structures in the motor neuron [3, 16]. Homozygous (*wr/wr*) mice exhibit progressive locomotor impairment with corresponding muscle atrophy, hyperreflexia, weakness, cramps, and ultimately respiratory failure caused by degenerative motor neurons in the spinal cord, brain stem, and motor cortex, whereas the (wobbler) WR mice show no clinical abnormalities during the first 3 weeks after birth (pre-symptomatic period) [17, 18]. At the age of 4 weeks to 3 months, the WR mice are conspicuous due to a reduced body weight, slightly unsteady gait, fine head tremor, and muscle atrophy, predominantly in the face and forelimbs [12, 18]. Life expectancy of homozygous WR mice varies from 120 days to up to 1 year, depending on the severity and progression of symptoms and the mouse strain-specific genetic background [16, 18]. The neurological phenotype highly resembles the symptoms of human ALS and offers some indication of a possible common pathway to cell death [15], even though there was no record of comparable VPS54 mutations in ALS patients until now [19].

## Methods

### Animals

C57BL/6-Vps54^**wr**^ homozygous for the Vps54 spontaneous mutation from a C57BL/6 background [14] was used in this study. The mice were kept at a constant room temperature of 22 °C ± 1 °C on a 12:12-h dark-light cycle with free access to food and water. The exact and special treatment of the homozygous wobbler mouse has been recently described [17]. With the aid of the mouse brain atlas Paxinos and Franklin, the neuronal tissue (layer V of the motor cortex; Bregma 2.46 mm to −1.34 mm) was dissected from the WR mice and contemporary wild-type mice (WT; C57BL/6) ranging in age from 20 to 60 days postnatal (d.p.n.) Observations were done on five WR mice and five WT mice per age group.

### Ethics statement

All protocols and experiments were performed under the terms of the German animal protection law and were permitted by the local authorities.

### Genotyping

Genomic DNA of an ear stamp (~2 mm diameter) was compound with 2 μl KAPA Express Extract enzyme and 10 μl 10× KAPA Express Extract buffer (Peqlab 07-KK7100-01, Erlangen, Germany), dissolved in 90 μl aqua destilata, and heated to 75 °C for 15 min to digest the stamps. Afterwards, the digestion enzymes were inactivated by heating the samples to 95 °C for 5 min. The wobbler (wr) and the wild-type (wt) alleles of the Vps54 gene in the mouse genome were determined by PCR employing the KAPA2G fast hot-start genotyping mix (Peqlab, 07-KK5621-01, Germany). For detection of the wr allele, we used the primers Vps54-wr-forward (5′-AGG CCT TAA AGA TCT GGA TCA-3′) and Vps54-wr-reverse255 (5′-TGC TCC TTA CTC AGG GAT GC-3′), whereas for the wt-allele we used Vps54-wt-forward413 (5′-GCT TCT CTG TTG AAG CCA CA-3′) and Vps54-wt-reverse (5′-CCC AGA TCT CGG CCA TAT TTA-3′) (Eurofin Genomics, Ebersberg, Germany). Amplification of DNA requires an initial DNA denaturation and polymerase activation step at 95 °C for 3 min, 35 cycles of DNA denaturation at 95 °C for 15 s, primer annealing at 63 °C for 15 s, and DNA elongation at 72 °C for 20 s. Afterwards, 1.5 % (*w*/*v*) Tris-borate-EDTA agarose gel electrophoresis was run at 130 mA for 30 min to separate the DNA fragments by size [17].

### Immunohistochemistry and immunofluorescence

The mice were perfused with 4 % (*w*/*v*) paraformaldehyde (PFA) as previously reported [17]. After post-fixation for 3 days, the brains were incubated in a cryo-protection solution (30 % (*w*/*v*) which is sucrose in phosphate-buffered saline (137 mM NaCl, 3 mM KCl, 10 mM Na_2_HPO_4,_ and 1 mM KH_2_PO_4_)) for 1 day. Subsequently, the brains were embedded and oriented in tissue freezing medium (Cryoglue, SLEE, Mayence, Germany) and were frozen and cut with a cryotome (Leica CM 3050 S, Wetzlar, Germany) in a chamber temperature of −24 °C, with the temperature of the microscope slide at −17 °C. The brain sections were collected on SuperFrost® Plus (Gerhard Mentzel, Braunschweig, Germany) slides and dried at 37 °C for 1 h [16]. Immunohistochemistry was performed on the 30-μm-thick sections of the mouse motor cortex and were washed (5′/wash) by phosphate-buffered saline (PBS) three times prior to incubation with 1 % (*v*/*v*) Triton X-100 at room temperature for 20 min to increase cell membrane permeability. Afterwards, the sections were washed five times (5′/wash) and incubated with various primary antibodies. Detection of activated microglial cells was performed with goat anti-Iba-1 (ab5076, Abcam, UK, 1:100) in conjunction with anti-goat IgG Cy3 (Sigma-Aldrich, Germany, 1:100) and mouse anti-neuronal nuclei (NeuN) (MAB377B, Chemicon, Germany, 1:100) in conjunction with anti-mouse IgG fluorescein isothiocyanate (FITC) (F1010, Sigma-Aldrich, 1:500). Furthermore, rabbit anti-TNF-α (ab34674, Abcam, 1:100) and rabbit anti-caspase 3, (966515, Cell Signalling, USA, 1:100) for indicating inflammation and apoptosis were used in conjunction with anti-rabbit IgG tetramethylrhodamine (TRITC) (t5268, Sigma-Aldrich, 1:500). Detection of reactive astrocytes was implemented with mouse anti-glial fibrillary acidic protein (GFAP) (G3893, Sigma-Aldrich, 1:100) and rabbit anti-S100 β (S2644, Sigma- Aldrich, 1:100) and depending on the primary antibody, treated with anti-mouse IgG FITC (F0257; Sigma-Aldrich, 1:500) and anti-rabbit IgG TRITC (t5268; Sigma-Aldrich, 1:500). All of the above primary antibodies have to be absorbed overnight at room temperature in a humidity chamber to avoid the rapid drying out of the specimens. Following five washes (5′/wash) with PBS and after incubation with the primary antibody, the samples were treated with 5 % bovine serum for 30 min to block non-specific bindings. Without washing the sections after this step, secondary antibody, as described above, was added for 2 1/2 h at room temperature. After washing five times (5′/wash) with PBS, the nuclear staining of the tissues was done by incubation with DAPI (B2261, Sigma-Aldrich, 1:1000) for 20 min. Finally, the sections were covered with fluorescence mounting medium (S3023, Dako, Germany) and evaluated with a Zeiss LSM510 Meta confocal laser scanning microscope.

### Electron microscopy

The mice were perfused with 2.5 % (*w*/*v*) glutaraldehyde (GA) as previously reported [17]. After post-fixation for 3 days in 2.5 % (*v*/*v*), the GA specimens were set in PB for 1 h and were washed in PB for 10 min in a following step. The samples of the WR and WT mice motor cortex were incubated with Dalton for 2 h and washed in PB for 10 min after that. Following that, the samples were dehydrate using an ascending concentration serial incubation in ethanol and were subsequently embedded in Epon in different ratios over night. The motor cortex specimens were cut with an Ultracut E Reichert-Jung, collected on Formvar coated grids, contrasted with uranyl acetate, and analyzed with a Philips EM 420 (Philips, Holland) transmission electron microscope with a digital CCD camera (Model 792 BioScan; Gatan, USA) and photographic plates system processed with a Ditabis Micron System.

### Statistical analysis

Quantitative analysis was done by confocal laser scanning microscopy (Zeiss LSM 510 Meta) in combination with Zeiss ×40 oil immersion lens (Plan-Neofluar, NA 1.3). Statistical analysis on the increase of microglial cells, TNF alpha, and caspase 3-positive neurons was performed by the use of 90 different slices. For each experiment, at least six different slices were used (WT: n 5; WR p20: n 5; WR p40: n 5; WR p60: n 5). The statistical significance was evaluated using the *t* test and t vert in Microsoft Excel.

## Results

### Microglial activation, up-regulated neuroinflammation, and worsened neuronal damage

**Fig. 2:**
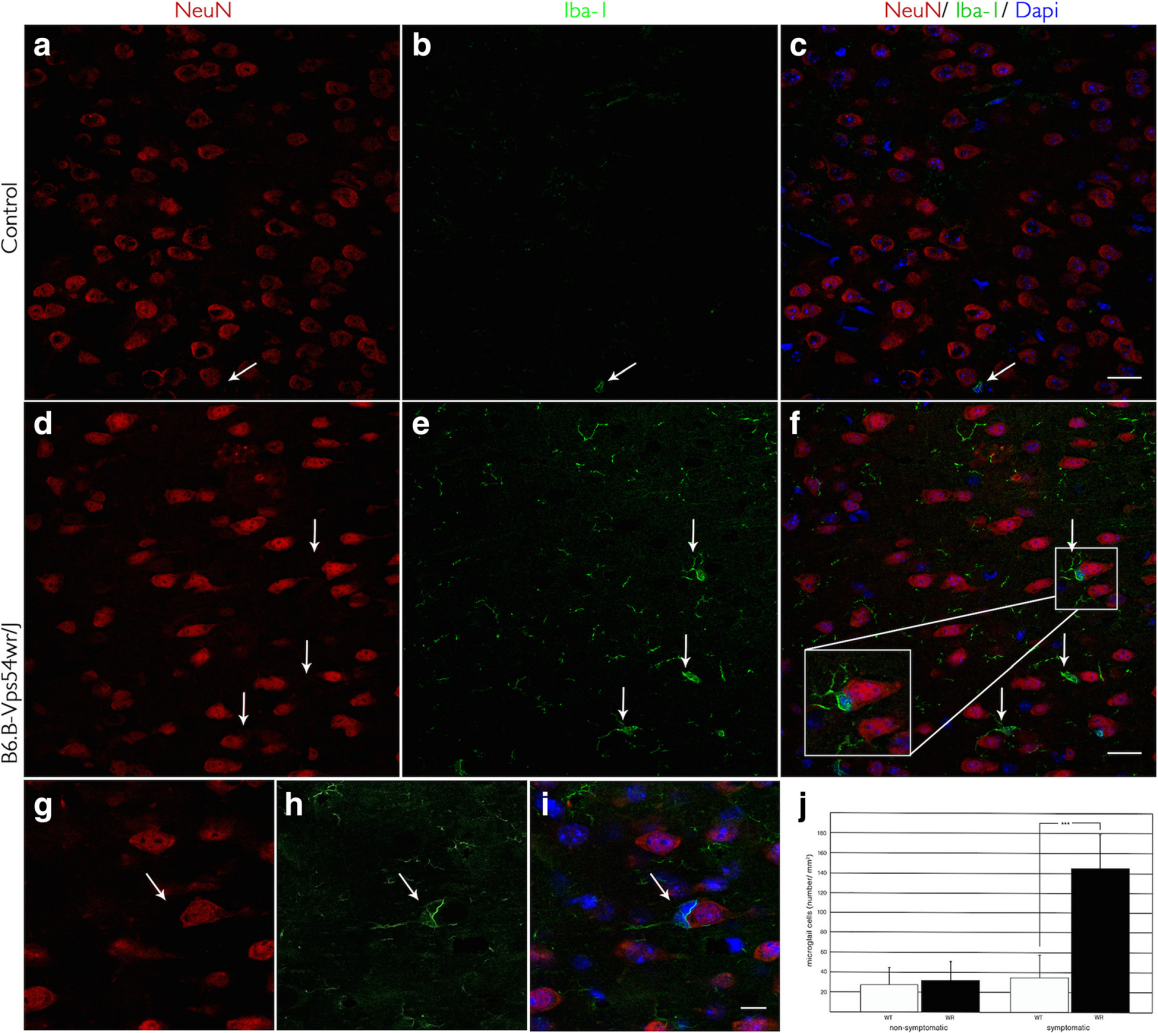
Visualization of Iba-1-labeled microglial cells in the motor cortex tissue of WR and WT mice 40 d.p.n. **a**–**c** Relation of activated microglial cells (*green*) and neurons labeled with neuronal nuclei antibody (*red*) in healthy brain tissue of WT mice. **d**–**f** Increased level of microglial cells (*green*) in affected and symptomatic WR mice. Magnification (**f**) shows the typical slender microglial processes (*green*) and the phagocytosis of a neuronal cell (*red*). *Scale bar* = 20 μm. **g**–**i** Phagocytosis of a degenerating neuron (*red*) by a microglial cell (*green*). Scale bar = 10 μm. **j** Increased number/mm^2^ of microglial cells in the motor cortex during the progression of the disease. *Error bars* represent SD ±

**Fig. 3:**
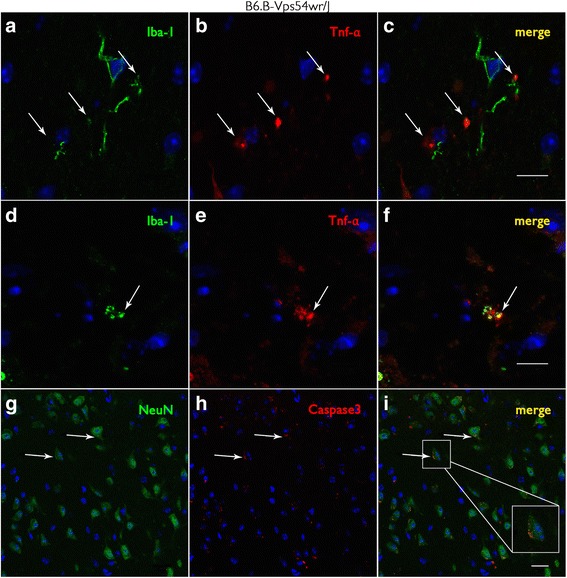
Tumor necrosis factor alpha (TNF-α) and Iba-1-labeled microglial cells and caspase 3-positive neuronal cells in motor cortex tissue of WR mice 40 d.p.n. **a**–**f** Iba-1-labeled microglial cells (*green*) also synthesize the cytokine TNF-α (*red*). **f** Microglial cells appear yellow due to the co-localization of Iba-1 (*green*) and TNF-α (*red*). *Scale bar* = 10 μm. **g**–**i** Caspase 3-positive (*red*) neurons labeled with NeuN (neuronal nuclei antibody) (*green*). Insert in **i** shows a caspase 3-positive neuron undergoing apoptosis. *Scale bar* = 20 μm

**Fig. 4:**
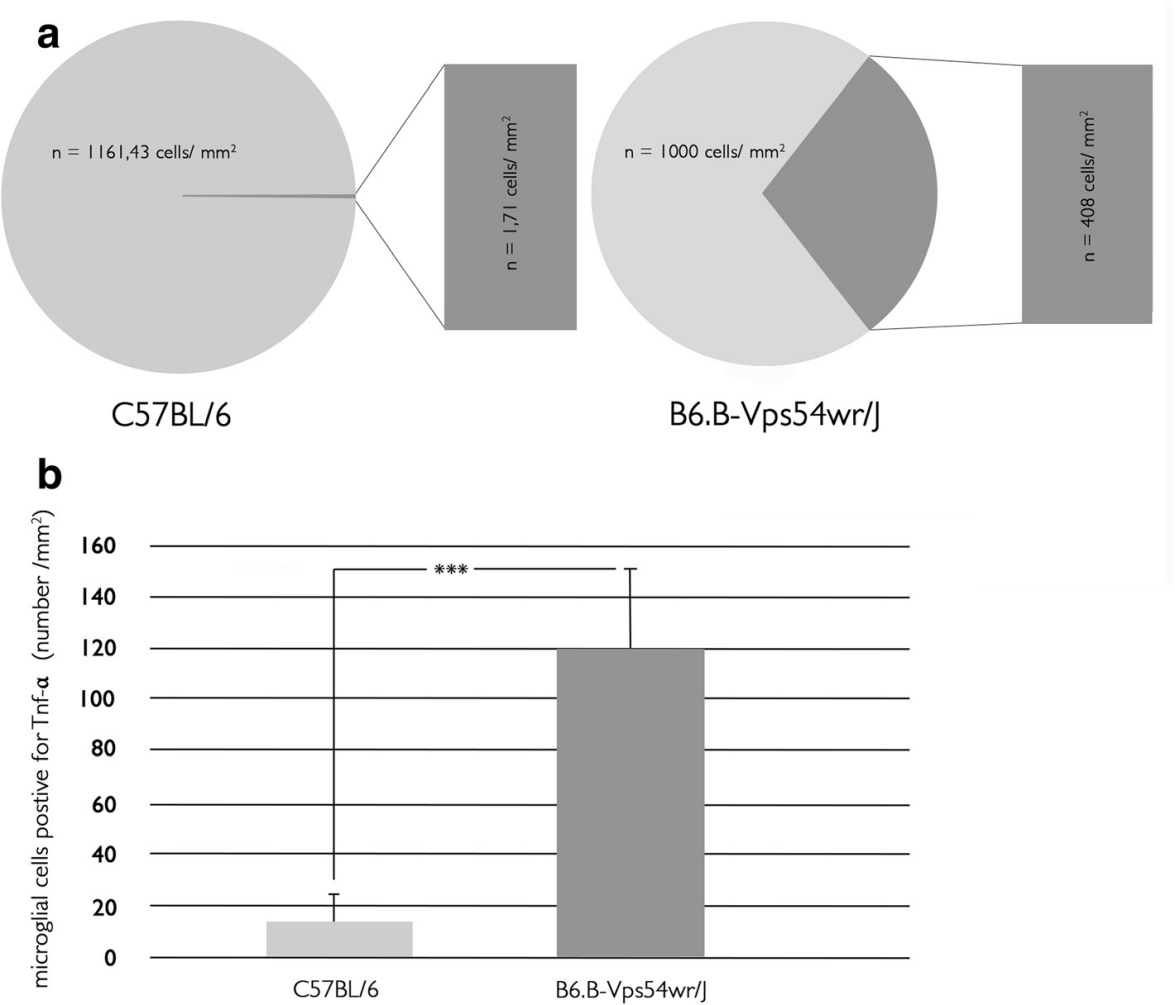
Quantification of caspase 3- and TNF-α-positive cells in the motor cortex of WR and WT mice. **a** A plot of density of caspase 3-positive neuronal cells in the motor cortex of WR and WT mice 40 d.p.n. **b** The number of microglial cells positive for TNF-α in the motor cortex of WR mice 40 d.p.n is significantly increased. Data are shown as mean ±SD; *p* < 0.001

Progression of glial activation is confirmed in this study by staining with the anti-Iba-1 antibody (ionizing calcium-binding adaptor molecule 1) in the motor cortex of the WR mice aged 20–60 d.p.n. Iba-1 is a calcium-binding protein whose expression is restricted to microglial cells [20] and reveals the highest increase in Iba-1-positive cells in the WR mice 40 d.p.n (Fig. 2, Additional file 1). Based on the intense reactivity of microglial cells in the WR mice, we predicted an up-regulation of the pro-inflammatory cytokine, TNF-α, whose level was also significantly increased 40 d.p.n (Fig. 3, Additional file 2). Further immunofluorescence study of the affected mice showed that cleaved caspase 3 was highly expressed in NeuN-positive neurons at approximately the same time of 40 d.p.n. (Fig. 3, Additional file 2). Caspase 3 is traditionally known as one of the primary effector caspases driving apoptosis and was not detected at any time in healthy WT control mice of equal age. Quantitative analysis gives a plot of density of caspase 3-positive neuronal cells in the motor cortex of the WR and WT mice 40 d.p.n. (Fig. 4a). Data show that on an average of 1161.43 neuronal cells/mm^2^ in the motor cortex of the WT mice, approximately 1.71 cells/mm^2^ are positive for caspase 3. In contrast, a significant increase in caspase 3-positive cells is seen in the motor cortex of the WR mice, with an average 408 neuronal cells/mm^2^ of 1000 neuronal cells/mm^2^. Besides this, an increased number of microglial cells positive for TNF-α in the motor cortex of the affected and symptomatic WR mice 40 d.p.n are detected (Fig. 4b). In comparison with that, the data show a significantly lowered quantity of TNF-α-positive microglial cells in the motor cortex of the WT mice 40 d.p.n.

### Reactive astrocytes

**Fig. 5:**
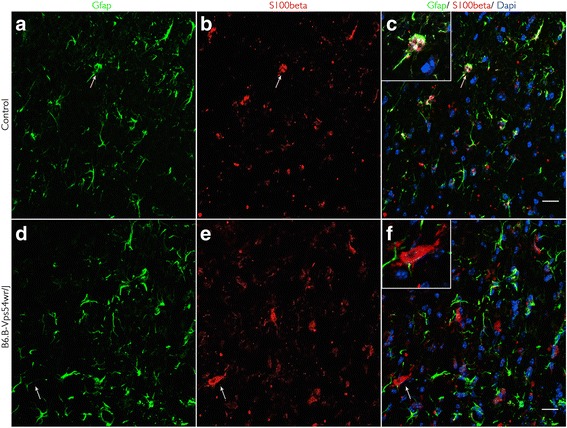
Identification of reactive astrocytes in motor cortex sections of WR and WT mice 40 d.p.n. **a**–**c** GFAP-labeled astrocytes (*green*) strictly co-localized (*magnification*) with S100 β protein (*red*) in the brain tissue of control mice in moderate concentration. **d**–**f** S100 β was up-regulated (*magnification*) in symptomatic mice and expressed in a population of hypertrophic astrocytes. *Scale bar* = 20 μm

Astrocyte activation in degenerating motor cortices was determined by using anti-GFAP and anti-S100 β immunoreactivity. S100 β is a calcium-binding protein mainly produced and located in the cytoplasm and nucleus of non-activated astrocytes. It exerts autocrine and paracrine effects on neurons and glial cells. Depending on its concentration and localisation, S100 β regulates the cytoskeletal structure and cell proliferation, stimulates the expression of pro-inflammatory cytokines, and may induce apoptotic cell death if over-expressed. The staining of astrocytes and S100 β was low or moderate in younger and asymptomatic mice (not shown) and increased dramatically during the symptomatic stage at the age of 40 d.p.n. (Fig. 5). Our results show a link between reactive astrocytes and the expression of the S100 β protein by co-localization in hypertrophic astrocytes. Astrogliosis and S100 β overexpression in the WR mice were typically observed nearby damaged neurons in the motor cortex, while astrocytes positive for GFAP in the contemporary WT mice were easily identifiable surrounding blood vessels, as shown in the spinal cord and brain stem [21].

### Activated microglia cells and degenerative neurons in ultrathin sections

**Fig. 6:**
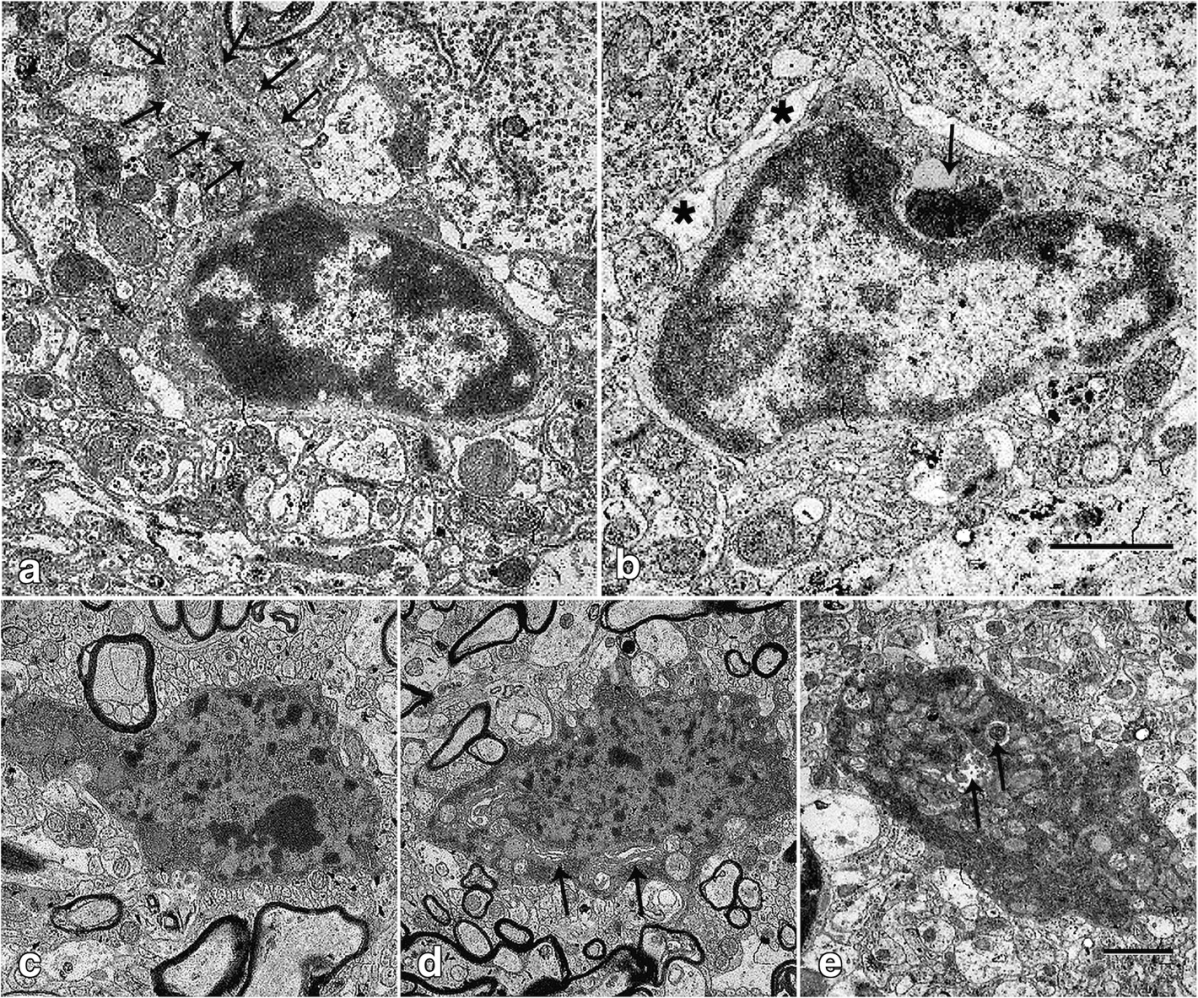
Ultrastructure of microglia and degenerating neurons in the motor cortex of B6.B-Vps54wr/J mice. **a**–**e** Electron micrographs from C57BL/6 J-*Vps54* ^*wr/wr*^ mice 40 d.p.n. **a** A typical microglial cell with dense heterochromatin lining the nuclear membrane, a narrow rim of contrasting light cytoplasm, and slender microglial processes (*arrows*). **b** Microglial cell containing debris of unknown origin (*arrow*). These morphologic features are associated with phagocytotic cells. *Asterisk*: tagged astrocytic process. *Scale bar* = 5 μm. **c**–**e** Transmission electron micrograph of degenerating neurons. They show an irregular morphology with highly electron dense cytoplasm containing dense vacuoles, swollen mitochondria, and a dilated ER-Golgi network (*arrow*). The nucleus displayed highly condensed chromatin, clustered in peripheral bundles. *Scale bar* = 20 μm

To confirm the previous results, we show a severe increase in activated microglial cells and aggravated neuronal damage in the motor cortex of the affected wobbler mice by using transmission electron microscopy (Fig. 6). Activated microglial cells display dense chromatin, forming a layer beneath the nuclear membrane, along with having a low density of cytoplasm. In most cases, these cells are characterized by large perikarya and slender microglial processes, which also contain degenerating material of unknown origin. In addition, degenerative neurons are detectable in the motor cortex of the wobbler mice, showing classic features of apoptosis as well as degenerative changes in cytoplasmic organelles such as mitochondria, rough endoplasmic reticulum, and a disorganized Golgi apparatus.

## Discussion

Our present study demonstrates an abnormal density of activated, i.e., morphologically modified, and up-regulated microglial cells in the motor cortex region of the WR mouse, as well as an abundance of tumor necrosis factor alpha and apoptotic caspase 3-positive neurons. Progressive neurodegeneration resulting in microglial activation and cell loss in the thalamus, cerebellum, and brain stem, for example, has already been discussed in previous studies [21], whereas the correlation of inflammation and advancing degeneration, as well as the mechanism by which neurons degenerate, still remains unresolved [22]. Several lines of research suggest an involvement of oxidative stress, excitotoxic mechanisms, and altered protein catabolism in the cascade of events leading to neuronal degeneration [23]. There is some evidence that activation of microglial cells occurred in response to acute neuronal degeneration and death [21] and also in chronic neurodegenerative diseases such as Alzheimer and Parkinson diseases [24]. An association between chronic inflammation and chronic neurodegeneration has been found in numerous investigations of Alzheimer disease, where activated microglial cells are closely related to amyloid beta deposits and show increased levels of tumor necrosis factor alpha [25]. Enlarged endosomal structures positive for amyloid precursor protein (APP) have already been identified in the WR motor neurons of the spinal cord and brain stem [3] and would be an interesting target for further analysis in the WR mouse motor cortex. Impaired vesicle trafficking might cause neuronal stress and chronic inflammation thereafter, similar to corresponding observations in Alzheimer disease. It remains to be seen whether this enhanced synthesis of pro-inflammatory mediators is responsible for an acceleration of the disease and the acute symptoms of chronic neurodegenerative disease or simply the logical consequence of neuron loss [24]. Based on this issue, some retrospective studies imply that the immediate treatment with anti-inflammatory drugs (i.e., non-steroidal anti-inflammatory drugs), or the blocking of signaling pathways, may slow the onset and progression of symptoms and positively influence the quality of life of chronically affected patients [24–26]. It would also be of value to investigate the role of inflammation in the case of amyotrophic lateral sclerosis as well. Our present data present an important insight into how microglial activation and inflammatory processes are associated with motor neuron death in a sporadic model of ALS and provide a clue for a novel therapeutic target that can be modulated to delay the onset and progression of this severe disease.

With the aid of immunohistochemistry, our study also proves the presence of reactive astrocytes, characterized by an up-regulated expression of GFAP and an increased immunoreactivity for calcium-binding protein S100 β [27] in the mouse motor cortex of the WR mice. S100 β may have toxic functions if overexpressed [28] and has been linked to the progression of neuronal pathology [27]. In the SOD1 mouse spinal cord, and in cases of ALS, astrocytes positive for S100 β were detected; however, the expression of S100 β was limited to just a few astrocytes in WT mice and human controls [27]. In addition, WR mice as well as human patients develop a typical gliosis with proliferative and hypertrophic astrocytes [29] in the spinal cord. This kind of reactive astrogliosis may be an important process leading to the formation of a glial scar that inhibits neuronal growth after a central nervous system injury to physically isolate the injured area [30, 31]. Additionally, recent studies have shown that specifically in ALS, astrocytes can induce oxidative stress and motor neuron death by producing numerous toxic molecules [6, 32, 33] such as nitric oxide or peroxynitrite [34]. It has been reported that WR astrocytes especially display a toxic effect on neuronal cells in the spinal cord instead of promoting neuronal outgrowth [35, 36] and thus might contribute significantly to the progression of the disease. In other advanced stages of neurodegenerative disorders, astrocytes show an increased secretion of TNF-related apoptosis-inducing ligand (TRAIL), binding to a death receptor and initiating apoptotic cell death [37].

The glial reaction is generally a well-known consequence of neuronal death in neurodegenerative diseases such as Parkinson’s disease. Post mortem examinations of patients demonstrated massive neuronal cell loss surrounded by a severe astrogliosis and an increase of activated microglial cells, therefore strongly supporting the hypothesis that the glial reaction may be deleterious for damaged neurons in Parkinson disease [23]. Additionally, a higher density of glial cells, pro-inflammatory cytokines, as well as caspase proteins have been reported in the brain and cerebrospinal fluid of Parkinson patients [38]. Activation of the TNF-α receptor transduction pathways seems to be of particular relevance for the progression of Parkinson disease by initiating caspase 3 and caspase 8 activation and thereby inducing apoptotic cell death.

Indeed, the glial reaction is not specific to Parkinson’s disease, rather, it is a common phenomenon in neurological disorders. Prolonged glial cell accumulation surrounding motor neurons in the spinal cord of wobbler mice, rats, and mice expressing the ALS-linked SOD1 mutation and even in post mortem studies is also well established to proceed the degeneration and subsequent paralysis of affected animals and ALS patients [34, 39, 40]. In particular, microglial activation in the spinal cord and brain stem of ALS patients and various SOD1 mutants has been reported in numerous studies, so that several groups had the idea to inhibit microglial activation by using minocycline, a tetracycline derivate [41]. With the aid of minocycline, they were able to slow down the onset and progression of disease in young mice [42], but it still remains uncertain what kind of cells is influenced by minocycline. Nevertheless, due to its beneficial effect, minocycline is a possible therapeutical strategy [43]. A further aspect observed in the mutant SOD1 mouse spinal cord, as well as what we have shown in the motor cortex of the wobbler mutant, is the up-regulated production of tumor necrosis factor in adult and symptomatic SOD1 and WR microglial cells compared to control microglial cells [44]. Up-regulation of the pro-inflammatory and neurotoxic tumor necrosis factor may also play a key role in progressive neurodegeneration and should be considered for use in an experimental therapeutic treatment strategy, possibly in terms of a TNF-α antagonist [45]. Overall, microglial cells show a direct toxic effect on the motor neurons of mutant SOD1 mice [46] and may be an important component in initiation of apoptotic cell death by TNF-α as described above. However, it is likely that the progressive neuronal degeneration is based on the interplay of various mechanisms. Inflammatory processes in the motor cortex of a sporadic model of ALS, as mentioned above, have not been described until now. This new context of an impaired motor cortex as well as involuntary movement provides another approach of effective therapy, because the rapid progression of the disease is not restricted to damaged neurons of the spinal cord, brain stem, and cerebellum. Earlier studies have shown that even the motor cortex lesions exert a considerable impact on the use of limbs, manual skill, speed of forelimb movement, fore- and hindlimb placing, reaching, forelimb strength, beam walking, spatial navigation, and spatial memory [47]. Thus, anti-inflammatory treatment targeting motor cortex areas might lead to beneficial effects for ALS patients.

## Conclusions

The main purpose of our research is to provide detailed insights into the role of neuroinflammation in the pathogenesis of the sporadic form of ALS. Inflammation, evidenced by the activation of microglia and astrocytes, is an essential hallmark of neurodegenerative disorders and might be a promising target of therapeutic intervention for ALS. Understanding the roles and relationships between microglial activation, pro-inflammatory molecules, astrocytes, and motor neurons is particularly important for attenuating neuroinflammation in the early stages of ALS, and thereby slowing the progression of the disease. Likewise, the proven inflammatory process of neurons in the motor cortex of the WR mice, that so far has not received much attention, could have an impact on the anti-inflammatory treatment of human ALS patients.

## Additional files

Additional file 1:
**Comparison between Iba-1-labeled microglial cells in the motor cortex of WT mice and intense symptomatic WR mice 60 d.p.n.** (A–C) Relation of activated microglial cells (red) and neurons labeled with neuronal nuclei antibody (green) in brain tissue of WT mice. (D–F) Relation of activated microglial cells (red) and neurons labeled with neuronal nuclei antibody (green) in the brain tissue of severely symptomatic WR mice. (Scale bar = 20 μm). (TIF 10223 kb) Additional file 2:
**Visualization of the weak staining of Iba-1- and TNF-α-labeled microglial and caspase 3-positive neuronal cells in motor cortex tissue of WR mice 20 d.p.n.** (A–C) Relation of activated microglial cells (red) and neurons labeled with neuronal nuclei antibody (green) in brain tissue of non-symptomatic WR mice. (D–F) Iba-1-labeled microglial cells (red) synthesizing the cytokine TNF-α (green). (G–H) Caspase 3-positive (red) neurons labeled with NeuN (neuronal nuclei antibody) (green). (Scale bar = 20 μm). (TIF 15107 kb)
