# Supplementary figures and images for: Inflammation and neuronal death in the motor cortex of the wobbler mouse, an ALS animal model
Source: J Neuroinflammation. 2015 Nov 24;12:215. doi: 10.1186/s12974-015-0435-0 (PMC4657283; doi:10.1186/s12974-015-0435-0)

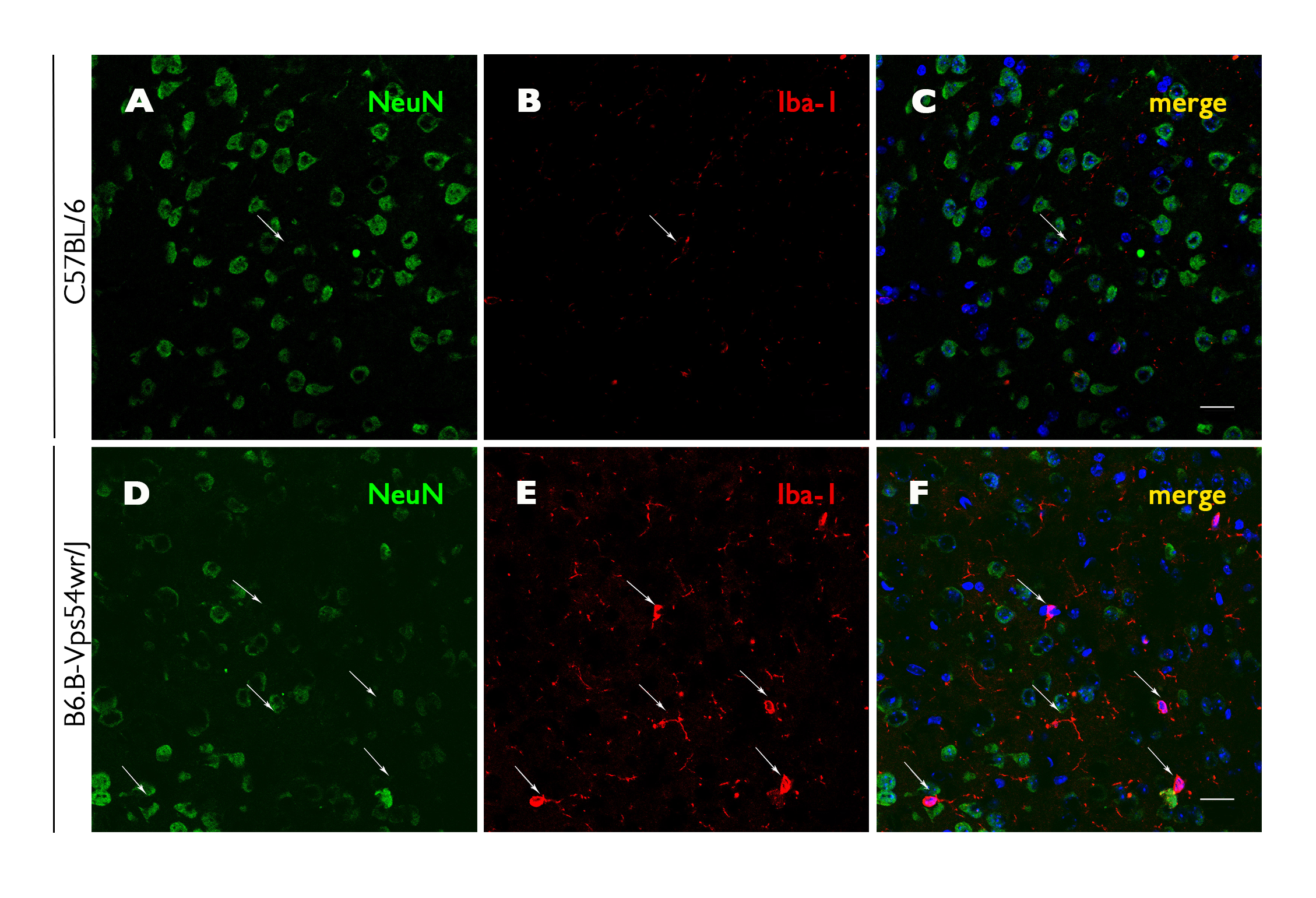

Supplement: Additional file 1: — Comparison between Iba-1-labeled microglial cells in the motor cortex of WT mice and intense symptomatic WR mice 60 d.p.n. (A–C) Relation of activated microglial cells (red) and neurons labeled with neuronal nuclei antibody (green) in brain tissue of WT mice. (D–F) Relation of activated microglial cells (red) and neurons labeled with neuronal nuclei antibody (green) in the brain tissue of severely symptomatic WR mice. (Scale bar = 20 μm). (TIF 10223 kb) [file 12974_2015_435_MOESM1_ESM.tif]

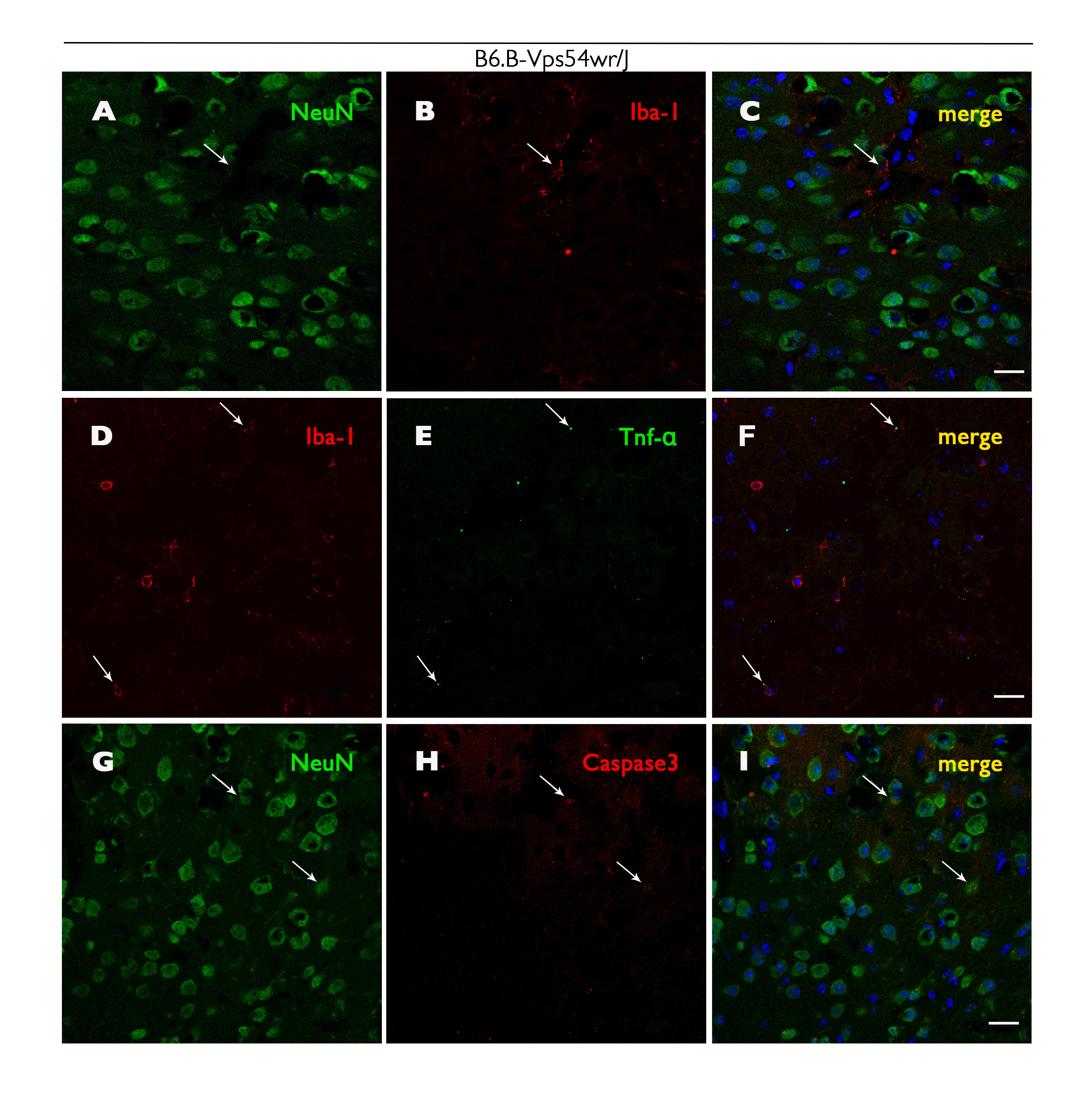

Supplement: Additional file 2: — Visualization of the weak staining of Iba-1- and TNF-α-labeled microglial and caspase 3-positive neuronal cells in motor cortex tissue of WR mice 20 d.p.n. (A–C) Relation of activated microglial cells (red) and neurons labeled with neuronal nuclei antibody (green) in brain tissue of non-symptomatic WR mice. (D–F) Iba-1-labeled microglial cells (red) synthesizing the cytokine TNF-α (green). (G–H) Caspase 3-positive (red) neurons labeled with NeuN (neuronal nuclei antibody) (green). (Scale bar = 20 μm). (TIF 15107 kb) [file 12974_2015_435_MOESM2_ESM.tif]
